# Extending MQTT with Real-Time Communication Services Based on SDN

**DOI:** 10.3390/s22093162

**Published:** 2022-04-20

**Authors:** Ehsan Shahri, Paulo Pedreiras, Luis Almeida

**Affiliations:** 1 Department of Electronics, Telecommunications and Informatics (DETI), University of Aveiro, 3810-193 Aveiro, Portugal; pbrp@ua.pt; 2 Instituto de Telecomunicações, Campus de Santiago, 3810-193 Aveiro, Portugal; 3 Research Center in Real-Time and Embedded Computing Systems (CISTER), University of Porto, 4200-135 Porto, Portugal; lda@fe.up.pt; 4 Faculdade de Engenharia da Universidade do Porto (FEUP), University of Porto, 4200-465 Porto, Portugal

**Keywords:** MQTT, IoT, IIoT, real-time communication, SDN, OpenFlow

## Abstract

MQTT is one of the most popular application-layer protocols used in the scope of the Internet-of-Things (IoT) and Industrial-Internet-of-Things (IIoT), given its suitability for resource-constrained embedded systems. However, MQTT Quality-of-Service policies do not support timeliness requirements, which is common in IIoT. The literature reports several research works that address this limitation, but they are limited in scope (e.g., improvements in the broker’s internal operation, control of the publisher’s data rate, and path optimizations). Conversely, this paper presents a comprehensive architectural approach, proposing a set of extensions to the MQTT protocol that allow applications to explicitly specify real-time requirements and instantiate corresponding network reservations to enforce the desired temporal behavior. Such reservations are enforced via Software Defined Networking, specifically the OpenFlow protocol, but other protocols that allow bandwidth reservations, e.g., TSN, can also be used. This paper presents the proposed system architecture together with extensive emulation and implementation results that validate the feasibility of the approach, showing that time-sensitive MQTT traffic can be effectively segregated and prioritized to meet application-defined real-time requirements. Using several combinations of network topologies and load levels and comparing to the absence of the proposed real-time mechanisms, both average and worst-case latencies of the time-sensitive traffic decreased to approximately half, while for the normal traffic, they increased by approximately 10%.

## 1. Introduction

Steady technology advancements continuously foster the Internet-of-Things (IoT), enabling a massive and unprecedented integration of digital devices without requiring explicit human interaction. The popularity of the IoT has expanded to a vast range of application domains, from smart grids [1] to industrial automation [2,3], medical systems [4], wearable devices [5], agriculture [6] and many others. This large diversity of application domains necessarily brings along an increase in requirements heterogeneity. Notably, Industrial IoT (IIoT), which is one of the pillars of Industry 4.0, brings along stringent requirements on real-time (RT) performance and reliability [7], needing an adequate computing and communication infrastructure.

Concerning communications, the Message Queuing Telemetry Transport protocol (MQTT) [8] is one of the most popular application-layer protocols used in the IoT, with a growing presence in the IIoT, too. Its popularity stems from its simplicity, low footprint, scalability, and effective publisher-subscriber messaging model, which fits resource-constrained devices. MQTT is normally used over TCP/IP networks, building on ordered and lossless bi-directional channels. One significant limitation of MQTT is that its Quality-of-Service (QoS) policies address exclusively message delivery, missing real-time requirements entirely. This limitation has been addressed by the scientific community, but the approaches available in the literature are of limited scope, focusing on the timing behavior of the broker, controlling the load submitted to the network, or on network path optimizations. Conversely, this paper proposes a new architectural approach together with a set of extensions to the MQTT protocol that allow applications to state their real-time requirements and translate those requirements to network reservations. The reservations are enforced using Software Defined Networking (SDN), particularly the OpenFlow protocol, but any network protocol supporting dynamic bandwidth reservations (e.g., IEEE 802.1 TSN) can potentially be used.

The remainder of this paper is organized as follows. Section 2 provides background on MQTT and SDN. Section 3 overviews the state-of-the-art, with a focus on improvements of MQTT real-time performance and use of SDN for supporting real-time services, highlighting the novelty of the approach herein presented. Section 4 introduces the proposed MQTT real-time extensions (RT-MQTT), followed by an extensive performance evaluation via emulation in Section 5. Section 6 presents an implementation of RT-MQTT on COTS hardware, showing its practicality. Finally, Section 7 presents the main conclusions and identifies lines for future work.

## 2. Background

This section briefly overviews MQTT and SDN, emphasizing the features that are more relevant to this paper. A short discussion about security aspects and their implications is also provided.

### 2.1. MQTT

MQTT [9] is a lightweight publisher/subscriber application-layer protocol suitable for low-bandwidth environments composed of resource-constrained end-nodes. The MQTT architecture comprises a broker and a potentially large number of end nodes designated as clients. The broker mediates all communications; thus, clients interact only with the broker. Clients can be data sources (publishers), data consumers (subscribers), or both. Data addressing is carried out via the concept of topics. Topics are coded in MQTT messages and are structured hierarchically, following an organization that resembles folders and files in a file system. Clients that want to receive updates on a given topic first have to register as subscribers of such a topic. Thus, when a client publishes a message, the broker receives it, identifies the corresponding topic, identifies the list of subscribers and forwards the publisher message to each one of them. As the broker mediates, all communications, publisher, and subscriber clients are decoupled both in space and time. This functionality, combined with the protocol simplicity, scalability, and low footprint, are among the chief reasons behind the popularity of MQTT.

MQTT provides three QoS levels, all associated with message delivery. QoS 0 (send once) is the lowest QoS level and messages are sent without acknowledgment, thus without delivery guarantees beyond those provided by the underlying TCP protocol. QoS 1 (deliver at least once) guarantees that a message is delivered at least once to its receiver, either broker or subscriber, but there can be duplicates due to retransmissions. QoS 2 (deliver exactly once) provides the highest QoS level, guaranteeing that each message is received exactly once by its receiver. The complexity and latency of these QoS levels naturally grow from QoS 0, the simplest and fastest, to QoS 2, which is the most resource-demanding and slowest one, using a four-part handshake between senders and the receivers. The QoS level of each link (publisher-broker and broker-subscriber) is set independently.

The latest version of MQTT (MQTT V5.0 [9]) introduces the so-called *User Properties*, which are particularly relevant for this work. *User Properties* are an extension mechanism that consists of an array of UTF-8 key/value pairs, allowing the addition of user-defined information to MQTT messages conveyed in the corresponding message property field. Hence, metadata associated with arbitrary user properties can be exchanged between publisher, broker, and subscriber. This is the mechanism used in this work to allow nodes to specify the real-time requirements of topics.

### 2.2. SDN

SDN is a network management paradigm that decouples network control from packet forwarding functionality [10], aiming at enabling a programmatic, dynamic, flexible, efficient, and simple to manage network configuration. The SDN architecture defines two layers, the so-called data plane, where SDN switches carry out packet forwarding, and the control plane, where a logically centralized controller manages the network. The controller has access to all switches via suitable management ports, thus having full knowledge of the network state, including the number and characteristics of devices, topology, and resource utilization. This information can be made available to applications that can both adjust to the network and modify the network configuration, e.g., requesting the controller to configure forwarding rules in the switches to attain the desired handling of data packets. The controller interface with the switches is called Southbound, while the interface with the applications is known as Northbound.

The OpenFlow (OF) protocol [11], standardized by the Open Networking Foundation (ONF), is the de facto SDN Southbound interface standard protocol for communication between OF controllers and OF switches. OF controllers can dynamically configure and manage a set of OF switches, instructing them on how to handle data packets. OF switches comprise one or more flow tables that contain a set of prioritized flow rules. In turn, flow rules have filters that allow identifying packets and actions that are applied to those packets, such as modifying a packet, forwarding it to a specific port, or dropping it. Thus, when a packet arrives at an OF switch, it is matched against the flow rules, and if a match is found, the corresponding actions are applied. If there is no match, a default action is applied, e.g., forwarding the packet to the controller, sending it to a group table, or dropping it. Group tables contain a subset of instructions similar to those of flow tables, with similar functions.

### 2.3. Network Security: Need and Issues

Factory automation commonly includes processes (e.g., cell control and synchronization) that exhibit latency and latency jitter constraints. Supporting these processes in a distributed fashion typically requires real-time protocols based on cabled physical media, particularly when the requirements are tight (e.g., in the ms range). Integrating these distributed real-time processes in broader scopes, e.g., production lines or aggregates of cells raises the need for security mechanisms given the additional threat surface. However, the overhead imposed by these mechanisms can conflict with the timeliness requirements. Given that the risk of security threats originating physically inside the factory premises is relatively low, security mechanisms are commonly discarded in inner segments of the system while relying on strong security protection in boundary routers or gateways.

Nevertheless, the current trend towards ubiquitous access and full integration of industrial networks with the Internet turns security mechanisms fundamental, even if at the expense of some degradation in timeliness and predictability, if that can be tolerated. For this reason, application-layer messaging systems (e.g., MQTT and AMQP) frequently embody security protocols, being SSL/TLS the state of practice in secure communication. In any case, real-time add-ons or adaptations of such application-layer messaging frequently include a node in the middle, e.g., a network manager or controller as in SDN, that requires clear-text access to the traffic payload for control purposes. This feature, frequently called Man-in-the-Middle, is not transparently compatible with SSL/TLS. The literature reports several mechanisms to sort out this apparent incompatibility. Having a node in the middle of connections with end-to-end security is similar to Network Monitoring Systems on the Internet, which frequently require access to the clear-text payload, e.g., deep packet inspection, beyond packets metadata [12].

The work we present in this paper was devised by targeting inner segments in industrial networks and dispensing security mechanisms. However, its architecture belongs to the class of systems using a node in the middle. Thus, security mechanisms can be adopted using appropriate techniques, as referred to in the previous paragraph. The specific adaptations depend on multiple dimensions that must be considered, from overhead to latency, manageability, transparency, security degradation, etc. This topic will be addressed in future work.

## 3. Related Work

This section overviews the most relevant contributions found in the literature regarding MQTT real-time performance and the use of SDN for supporting real-time services.

### 3.1. On the Timeliness of MQTT

Tachibana et al. [13] propose a priority control mechanism for heterogeneous remote monitoring IoT systems based on MQTT. In their architecture, a Priority Broker controls the communication link utilization according to application requirements. Communication comprises three phases, namely registration, prioritized data exchange, and release. In the registration phase, end nodes register their profiles in an application server, together with associated QoS requirements. In the second phase, the Priority Broker collects this information and controls the end nodes sending time and rate using explicit authorization. The release phase closes the application connection, releasing the resources used. The paper reports relevant latency reductions and an increase in the successful sending ratio, which are proportional to the messages’ priority.

p-MQTT [14] aims at providing timely and reliable delivery of emergency events in IoT applications. To this end, p-MQTT provides prioritization for emergency events in the MQTT broker, which comprises three components: classification, virtual queues, and priority control. Published messages are inspected by the classification component and are placed, according to the message type field, in one of the prioritized virtual queues (Normal, Critical, and Urgent). The queues are then processed by the priority control component according to their relative importance. Kim et al. [15] follow a similar approach, proposing a prioritization system that employs a two-bit priority field placed on the fixed MQTT message header, providing four priority levels, ranging from no priority to urgent. The broker checks the priority field of each message, processing it accordingly. Both papers report a significant reduction in message latency, again proportional to the message priority.

Several other papers in the literature mention the use of MQTT in real-time applications, e.g., [16,17,18,19]. However, these works focus on platform or software aspects only, not supporting the explicit specification of real-time communication requirements nor exerting network control, thus limiting the attainable real-time performance.

### 3.2. Real-Time Traffic Support Using SDN

The scientific literature includes several works that aim at supporting real-time communications with Software-Defined Networking, particularly using the OpenFlow Southbound protocol. For example, the work in [20] proposes an OF controller, named OpenQoS, that supports multimedia delivery with bounded end-to-end latency. This framework differentiates between regular data and multimedia traffic using suitable filters applied to packet headers. Regular data packets are handled by standard routing mechanisms, namely shortest path routing, while multimedia traffic routing is set as a Constrained Shortest Path problem, solved to minimize a cost function while satisfying a given maximum delay. This QoS-oriented routing scheme is recomputed whenever there is a topology change. HiQoS [21] is another framework that uses multi-path routing but goes one step further by also using the queuing mechanisms of SDN switches to provide different bandwidth guarantees through each selected path.

Tomovic et al. [22] follow an approach similar to the Integrated Services (IntServ) Internet QoS model, offering hard QoS guarantees using bandwidth reservation and admission control. Flows can be *best-effort* or *QoS-guaranteed*. The controller performs constrained shortest path routing for QoS-guaranteed flows, reserving bandwidth along their path if a feasible one is found, otherwise rejecting the flow. Papers [23,24] propose a QoS framework that uses two queues to segregate high-priority from best-effort traffic, reserving bandwidth for the high-priority one. Kumar et al. [25] introduce an SDN-based framework to reduce the latency of real-time flows used in safety-critical and delay-sensitive applications. This framework segregates flows in different queues and implements bandwidth reservations to provide stable and bounded end-to-end delays. Jochen et al. [26] also present an SDN-based framework that provides end-to-end real-time communication services. Flows are allocated to different priority queues in each hop, optimizing the resource usage while satisfying delay and bandwidth constraints.

Celenlioglu et al. [27] present a routing and resource management model for SDN-based intra-domain networks focusing on scalability. They assume a set of logical paths between each ingress and egress switch, forming a virtual network. The controller manages these virtual networks online, using the edge switches and the pre-defined paths. Whenever a new flow arrives, the controller assigns the new flow to one of the virtual paths. Park et al. [28] also addresses scalability, aiming at reducing delay and bandwidth utilization in massive IoT MQTT-based applications, but missing real-time guarantees. The proposed framework establishes bidirectional SDN multicast trees between publishers and subscribers, bypassing the broker, thus avoiding a corresponding potential bottleneck. PrioSDN-Resource Manager [29] is a resource management framework relying on admission control for virtualized SDN-based networks combining SDN and Network Virtualization (NV), namely slices, to reduce the network management complexity under the varying workload and flow priorities. PrioSDN-RM allocates resources using a priority-based run-time bandwidth distribution mechanism. Whenever a new flow is generated by an end-node, the controller uses a priority-based admission control (PAC) module to calculate and allocate enough bandwidth for the flows of the slice.

All these approaches report significant improvements crucial to industrial applications, including latency reduction, higher determinism, and efficient bandwidth use, confirming that SDN can be effectively used in the industrial domain.

### 3.3. Novelty of the Proposal

The state-of-the-art review reveals a clear interest in improving the real-time performance of MQTT as well as the capacity of SDN to support real-time communications. Table 1 summarizes the main features of the related works analyzed in Section 3. This table also shows, however, that there is no integrated solution that allows to specify real-time requirements at the application layer and translate them into robust and effective network reservations able to guarantee traffic segregation and prioritization. On the one hand, works that have roots in MQTT are in many cases restricted to the internal operation of the broker using prioritized traffic handling [14,15]. When some kind of network control is exerted, it is restricted to the load submitted by publishers [13]. In this case, non-MQTT traffic can compromise the temporal behavior of the time-sensitive one. On the other hand, there are several relevant contributions regarding the use of SDN to support real-time communications [20,21,22,23,24,25,26,27], including approaches such as path optimization, flow prioritization, and bandwidth reservations. Despite providing robust solutions to protect time-sensitive traffic, these approaches lack integration with MQTT, thus preventing MQTT applications from interacting with the network layer to carry out the resource reservations necessary to meet their requirements.

Two recent contributions deserve a special remark. Park et al. [28] propose the DM-MQTT protocol, which is the only work that compares directly to RT-MQTT in the sense that it integrates MQTT and SDN. However, the focus of this work is on reducing bandwidth utilization in large-scale IoT deployments by providing a multicast mechanism that bypasses the MQTT broker. Traffic prioritization, admission control, and real-time guarantees are not addressed in this approach. It should be noted that the focus of RT-MQTT is not in such large-scale deployments but instead in more restricted setups, e.g., the ones found in factory or process automation domains, where the coexistence of heterogeneous traffic types is common, some of which with strict timeliness requirements, and where MQTT is gaining momentum. On the other hand, Fontes et al. [30] propose real-time extensions to MQTT-SN, a derivative of the MQTT protocol tailored to sensor networks. Despite supporting real-time requirements through network reservations, this work builds on specific MQTT-SN mechanisms that are not available in MQTT V5.0.

Therefore, to the best of our knowledge, the framework proposed in this paper improves the state-of-the-art in two main aspects:

- The use of MQTT v5.0 *User Properties* to specify real-time requirements for time-sensitive traffic, preserving full compatibility with standard MQTT devices and software components;
- The development of a resource management framework, which interfaces MQTT with the underlying networking infrastructure, that transparently creates and manages real-time communication channels according to the application requirements.

## 4. MQTT Real-Time Extensions

As referred before, the objective of this work is to allow the specification of real-time communication requirements for time-sensitive (TS) MQTT flows while preserving compatibility with the MQTT standard. This is achieved by resorting to the *User Properties*, available in MQTT V5.0 specification [9] to convey the real-time requirements of a given TS topic. This information is decoded by a Network Manager that then reserves appropriate communication channels for such flows. In this paper, we adopt SDN/Openflow to develop such a Network Manager and enforce network reservations, but potentially any other networking technology able to provide bandwidth reservations and traffic prioritization could be used, e.g., IEEE TSN [31] or HaRTES [32].

### 4.1. System Architecture

**Figure 1:**
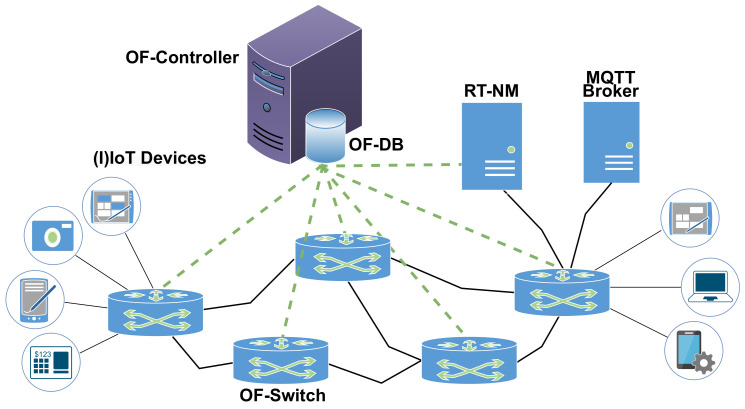
High-level RT-MQTT system architecture.

Figure 1 shows the proposed Real-Time MQTT (RT-MQTT) system architecture, including a set of OpenFlow switches (OF-Switches) connected to a central OpenFlow controller (OF-Controller), (I)IoT devices as MQTT clients, and an MQTT broker. Additionally, the system also includes a Real-Time Network Manager (RT-NM), which is logically placed between MQTT clients and the broker. The real-time requirements of all TS flows are kept in the OF-DataBase (OF-DB). Currently, the OF-Controller is based on the RYU framework [33] and the OF-Switches are instantiations of the *Open vSwitch* soft switch.

The RT-NM inspects all MQTT messages directed to the broker. When such messages convey real-time reservations, coded in the *User Properties* field, it registers the corresponding attributes, processes them, and updates the OF-DB in the OF-Controller. In turn, the OF-Controller updates the flow tables of the OF-Switches, creating real-time channels that match the specified real-time requirements.

### 4.2. Reservation Mechanism

As specified by the OpenFlow protocol, the OF-Controller is connected to all OF-Switches and holds a global view of the network. More specifically, the OF-Controller can collect detailed topological information, identifying all OF-Switches, ports, and links between them. In addition, OF-Switches are configured to send to the OF-Controller packets that do not match any installed flow rule via the so-called *PacketIn* messages. When the OF-Controller receives one of these messages, it checks the header fields of the corresponding incoming packet, getting information such as the source and destination IP address and the switch port at which it was originally received. Therefore the OF-Controller keeps a holistic network view that is then used for routing purposes.

The routing scheme adopted in this work is based on the Depth-First Search algorithm [34], with the link cost set as a weighted function of transmission delay and maximum available bandwidth of each possible path between source and destination nodes. The routing procedure is out of the scope of this paper and further details can be found in [35].

In general, it is not possible to find exclusive paths for each TS MQTT flow; therefore, these packets may share links with non-TS MQTT flows and other generic data sources. Consequently, without specific mechanisms, the potentially non-deterministic nature of other data sources would compromise the timeliness of TS MQTT flows. To address this problem, MQTT clients (both publishers and subscribers) associated with each time-sensitive flow communicate the corresponding real-time requirements via the MQTT *User Properties* field. RT-MQTT adopts the following subset of attributes, commonly used in real-time systems:

(1) $$F_{i}^{TS}=\left\{P_{i},T_{i},D_{i},B_{i},C_{i}\right\}$$

where:

*i*: flow identifier;
$P_{i}$: flow priority;
$T_{i}$: period or minimum inter-arrival time between two successive publish messages (by the publisher);
$D_{i}$: deadline, defined as the maximum allowed amount of time between the transmission (publisher to broker) and the reception (broker to subscriber) of a message;
$B_{i}$: maximum link bandwidth use;
$C_{i}$: maximum message payload size;

The RT-NM module intercepts all messages exchanged between the MQTT Broker and clients, extracting, when present, the real-time requirements associated with each flow ($F_{i}^{TS}$), which are then stored in a system’s real-time requirements table (SRT) that resides on the OF-DB. This table also holds the addresses of publishers and subscribers, which are obtained by the RT-NM module from the standard MQTT messages exchanged between the broker and clients during the connection set-up phase. More formally, the SRT is defined as follows:

(2) $$SRT=\left\{Pa_{i},\left\{Sa_{i,k}\right\},F_{i}^{TS}\right\}$$

where:

*i*:
flow identifier;
$Pa_{i}$:
address of publisher node;
{$Sa_{i,k}$}: set of *k* subscriber nodes addresses.

The information contained in the SRT, in conjunction with the topology information obtained by the OF-Controller, is then used to configure the real-time channels of all links in the path between the publisher and the Broker and between the Broker and the subscriber(s).

Note that this basic architecture can be complemented, if necessary, with control services. For example, changing the real-time attributes of a given flow can be restricted to a subset of (trusted) nodes, ignoring requests that originated elsewhere. Similarly, allocating resources to each flow can be constrained to (pre)defined bounds. These topics are out of the scope of this paper and will be addressed in future work.

**Figure 2:**
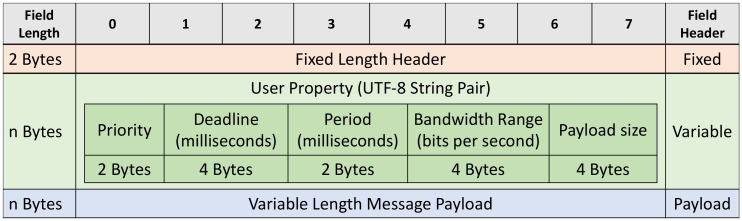
Structure of real-time attributes specification in RT-MQTT.

The message format used in RT-MQTT follows the MQTT V5.0 message structure, in which the *User Properties* field is placed in the Variable Header and conveys the relevant real-time attributes, as illustrated in Figure 2.

**Figure 3:**
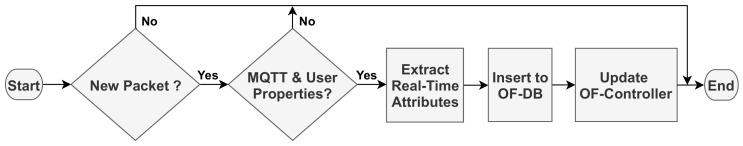
RT-NM operation flow diagram.

The core functionality of the real-time extensions is handled by the RT-NM. This module intercepts all messages from the MQTT clients to the broker, thus gathering the real-time attributes of time-sensitive traffic. As illustrated in Figure 3, when the RT-NM receives an MQTT client message, it inspects its content to determine the presence of a real-time reservation request, and, if one is found, the relevant real-time information is extracted and inserted into the OF-DB.

**Figure 4:**
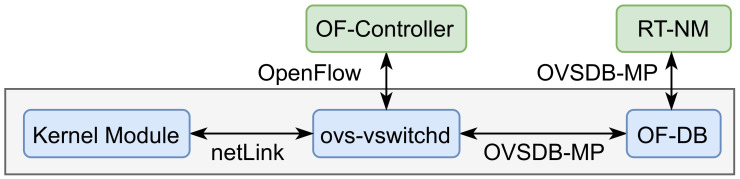
Network configuration process.

The interface between the RT-NM and the OF-DB is carried out using the OVSDB management protocol (OVSDB-MP) [36], as sketched in Figure 4.

Whenever the OF-DB is updated, the ovs-vswitchd daemon, which manages and controls the Open vSwitch switches, retrieves the real-time information. Then, in cooperation with the OF-Controller, it analyzes the set of registered real-time attributes to update the OF-Switch flow tables and set up the data paths. The ovs-vswitchd daemon also communicates with the kernel module of the corresponding node over netLink, a Linux kernel interface, to execute the associated actions corresponding to each received packet.

**Figure 5:**
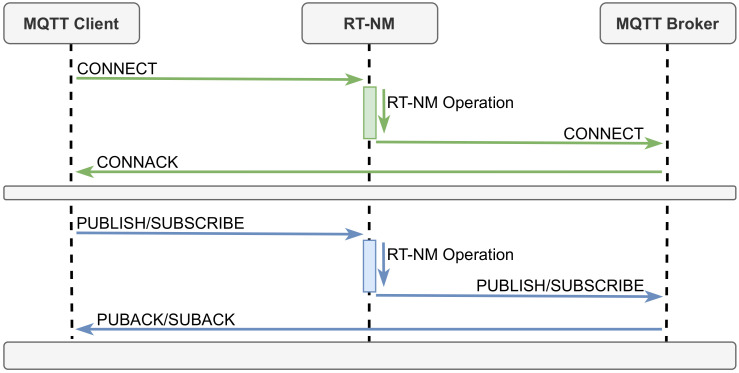
Message set up sequence diagram.

The real-time information can be modified at any time by MQTT clients, which can register or update real-time attributes of any given topic in the OF-DB. These attributes can be set initially, during the connection phase, using the CONNECT message, or added/updated later on, e.g., when a client publishes data via a PUBLISH message. In the same way, subscribers can specify real-time requirements when connecting or when subscribing to a topic using a SUBSCRIBE message. Figure 5 shows the sequence diagram for connecting, publishing, and subscribing. Note the RT-NM receives the messages, validates their attributes, and performs the corresponding network configuration, while the broker eventually sends an acknowledgment back to the client. A technical description of RT-MQTT services is publicly available (https://new-rt-mqtt-extension-api.readthedocs.io/en/latest/, accessed on 31 January 2022).

## 5. RT-MQTT Performance Assessment

The RT-MQTT protocol was instantiated on the Mininet emulation framework to validate its feasibility and assess its performance in multiple scenarios of different complexity.

### 5.1. Emulation Setup

We used the Mininet virtual network emulator, version 2.3.0d6 http://mininet.org/, accessed on 31 January 2022, together with Eclipse Mosquitto [37] (v2.0.10) and Eclipse Paho MQTT library to create MQTT clients and brokers. Mininet is executed on a laptop computer featuring a 4.9 GHz Intel Core i7 processor and 16 GB of RAM.

In the emulation experiments, the QoS of all MQTT messages is set to 1 (deliver at least once). This QoS level favors reliability over timeliness given its positive acknowledge and retry mechanism, and it was used since fault-tolerance is important for many IIoT applications. However, this is not expected to have a significant impact on cabled Ethernet networks, as we are currently using, given their low error rate.

To enforce traffic segregation and prioritization, we allocate a specific queue to each time-sensitive topic (Queues 1,2,…), with a higher queue number meaning higher priority. To flatten the impact of the topic size, we randomize the respective message length between 20 B and 2000 B in each experiment run. The queues are created by the RT-NM following *User Properties* sent by time-sensitive clients. The remaining bandwidth in each link is allocated to Queue 0 that receives the normal (non-time-sensitive) traffic. We generate time-sensitive and normal traffic in equal proportion.

The operational environment included heterogeneous data exchanges mimicking the diversity of industrial scenarios created with the *Distributed Internet Traffic Generator* (D-ITG) http://traffic.comics.unina.it/software/ITG/, (accessed on 31 January 2022) for TCP packets, *vsftpd* to transfer files using the File Transfer Protocol (FTP)) https://linuxconfig.org/how-to-setup-and-use-ftp-server-in-ubuntu-linux, (accessed on 31 January 2022) and *VLC media player* to generate audio/video streams. The bandwidth used by these traffic sources was limited to 10 Mbit/s, 32 kbit/s, and 800 kbit/s for D-ITG, VLC, and vsftpd, respectively. These are all non-real-time sources, thus assigned to Queue 0.

**Figure 6:**
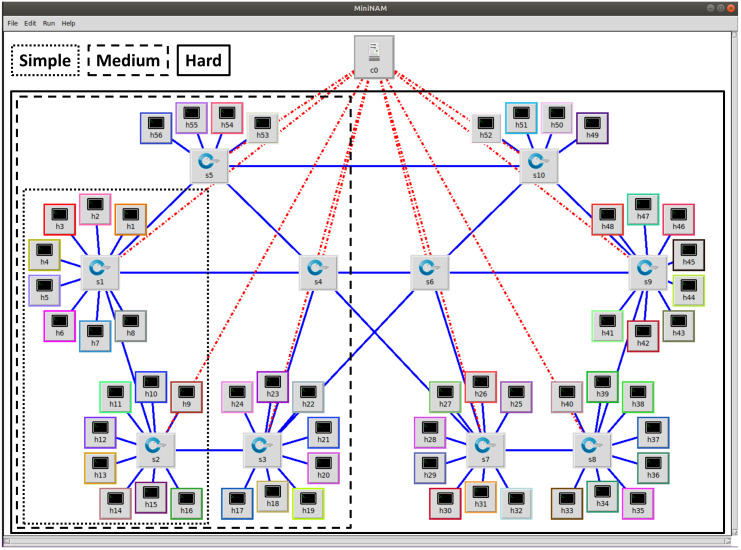
Network topologies used in the experiments.

The experiments consider three network topologies with different levels of complexity, named Simple, Medium, and Hard (Figure 6). These topologies comprise 2, 5, and 10 OF-Switches, respectively, with all links having 100 Mbit/s capacity. For each topology we generate three different load levels, labeled A, B, and C, consisting of 4, 10, and 14 MQTT publishers, each publishing data to one time-sensitive topic. In all topologies and load cases, we have multiple flow sharing links, either in the broker, in the subscribers, or in the inter-switch links along the flows paths. Publications by MQTT clients are not synchronized, following a 20 ms nominal period plus or minus a random uniform value within [00.4] ms, thus generating varying interference patterns. Table 2 summarizes the more relevant parameters of the emulation experiments.

The Simple topology includes the OF-controller (c0), two switches (s1 and s2), 13 MQTT publishers (h1 to h13), 2 MQTT subscribers (h14 and h15), the MQTT broker and the RT-NM (both in h16). Node h1 publishes in a topic without subscribers, just to increase the load in the broker uplink. All other topics are subscribed by one subscriber, only, either h14 or h15. The non-MQTT traffic includes D-ITG data from h4 to h12, VLC streams from h6 to h15 and *vsftpd* data from h7 to h16.

The Medium topology includes five OF-switches (s1 to s5) connected to the OF-controller. In this topology the MQTT subscribers are hosted in nodes h21 and h22, the MQTT broker and RT-NM are hosted in h23, and the remaining nodes host MQTT publishers. D-ITG sends data from h4 to h14, VLC from h6 to h19 and *vsftpd* from h7 to h24. h1 is similar to the previous case.

The Hard topology includes ten OF-switches (s1 to s10). Subscribers are now hosted in h36 and h46, the MQTT broker and RT-NM are hosted in h48, and the remaining nodes host MQTT publishers. D-ITG transmits data from h4 to h16, VLC from h6 to h34 and *vsftpd* from h7 to h49. h1 is also similar to the previous cases.

### 5.2. Experimental Results

**Figure 7:**
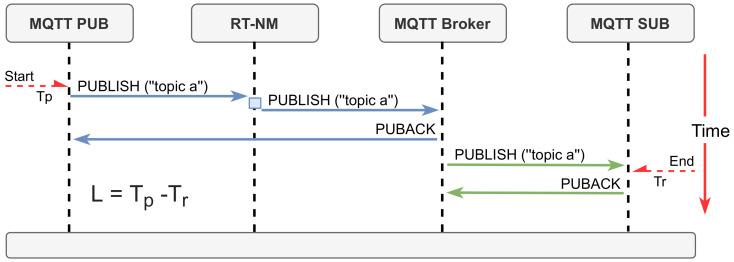
Latency measurement (L) in the RT-MQTT experiments.

We focus the experiments on the latency, defined as the time that each publication takes to travel from a publisher to the corresponding subscriber, measured at the network interface, thus including messages transmission times and software stack overheads (Figure 7).

The experiments tested all combinations of topology {Simple, Medium, Hard} and load levels {A, B, C}. Each combination was executed 200 times, with the location of each publisher (switch and port) randomly generated in each run.

**Figure 8:**
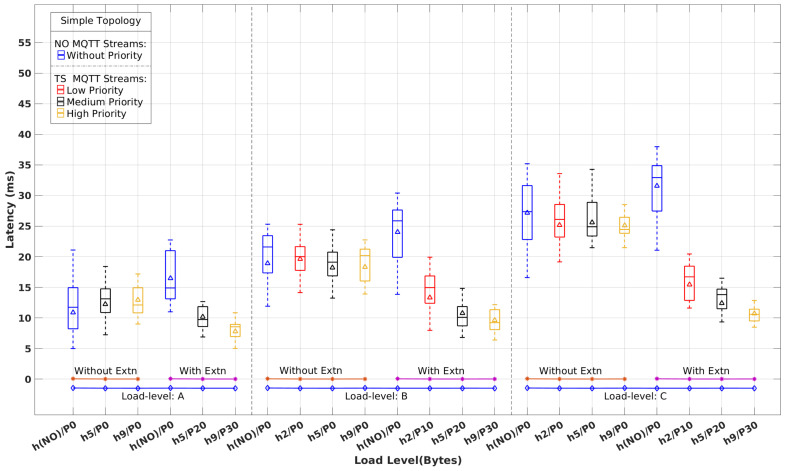
Latency of time-sensitive vs. normal MQTT flows with and without real-time extensions for the Simple topology and load levels A, B, and C.

**Figure 9:**
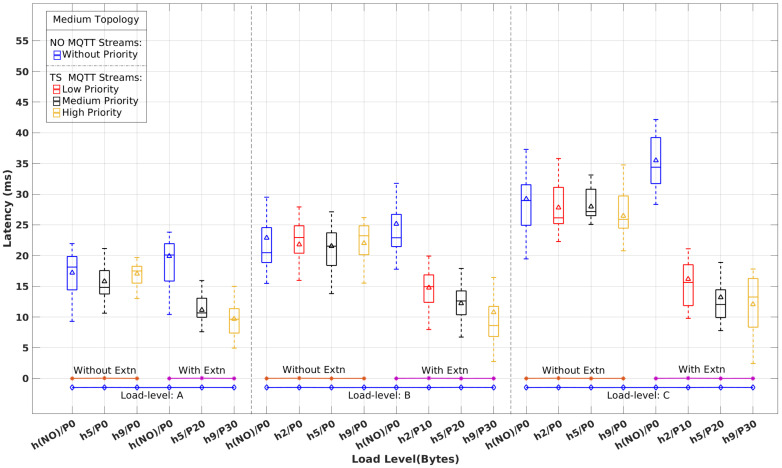
Latency of time-sensitive vs. normal MQTT flows with and without real-time extensions for the Medium topology and load levels A, B, and C.

**Figure 10:**
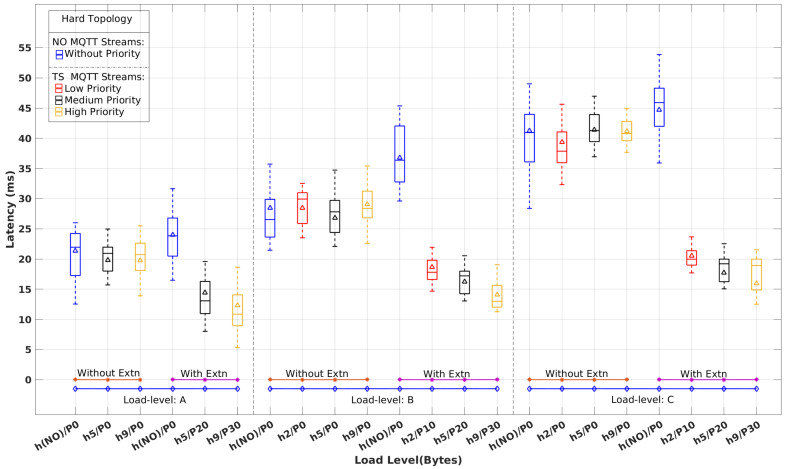
Latency of time-sensitive vs. normal MQTT flows with and without real-time extensions for the Hard topology and load levels A, B, and C.

Figure 8, Figure 9 and Figure 10 show the latency distribution with box plots that were obtained for normal (NO) and time-sensitive (TS) MQTT flows, with and without the real-time extensions, for each topology and all load-levels. As there is no differentiation among the NO flows, their latency values are aggregated in a single column. Concerning the TS flows, for the sake of clarity, we show just the ones with the highest, median, and lowest priority, except for load level A, which has two TS publishers, only, both shown in the figures. The horizontal axis identifies publishers, labeled as h#/P# for host h# with priority P#. {h(NO)} and P0 stand for the aggregation of NO MQTT flows.

Without the RT extensions in place, TS MQTT flows are handled by the network without any differentiation with respect to NO MQTT streams and the remaining background traffic. Therefore, we expect to see latency figures with similar patterns. This behavior is clearly observable in Figure 8, Figure 9 and Figure 10, which exhibit similar latency variations with the load level and the complexity of the topology, i.e., the number of hops crossed.

When the RT extensions are applied, we expect to observe the effects of segregation between TS and the remaining traffic, as well as the impact of the prioritization among distinct TS streams. Once again, these expectations are clearly confirmed in Figure 8, Figure 9 and Figure 10. In all scenarios, we observe a latency reduction of about one-half on the TS flows compared to the NO flows when the real-time extensions are in place. This reduction is slightly stronger for higher load levels in all topologies, as expected, too. Among the TS flows, we can also observe the consistency of the latency variation with the priority differentiation. On the other hand, the latency of the NO flows increases slightly, around 10%, when compared to the absence of the real-time mechanisms.

Table 3 shows the average and maximum latency values for the TS and NO flows represented in Figure 8, Figure 9 and Figure 10, to quantify the impact of the real-time extensions. One interesting observation is the reduced impact of the load level and topology complexity on the TS flows’ latency when compared to the NO flows. For example, varying the load level from A to C in the Simple topology causes the Avg(Max) latency of h5/P20 (intermediate priority) to grow from 10(13) ms to 12(16) ms while for h(NO)/P0 the variation is from 16(23) ms to 31(38) ms. This difference is even more pronounced in the Hard topology. Considering the highest priority TS flow h9/P30, the latency varies from 13(19) ms to 16(21) ms as opposed to 24(31) ms to 45(54) ms for h(NO)/P0.

Table 4 reports the Confidence Intervals (CIs) expressed by their Upper Bound (UB) and Lower Bound (LB) for a confidence level of 99% for the multiple combinations of topologies and load levels. The CI bounds, as expected, also increase with both growing load level (left to right) and growing network complexity (top to bottom). For the same configuration (topology and load level), higher priority levels have lower CI bounds, following the overall latency trends. Finally, the CI width, i.e., the difference between the upper and lower bounds (UB-LB), is significantly larger for the NO traffic when compared with the TS traffic. This is also expected as the NO traffic is subject to higher interference levels, both mutual interference and caused by the TS traffic, resulting in higher dispersion.

## 6. RT-MQTT Hardware Implementation

To further validate the practicality of RT-MQTT and confirm the emulation results presented before, we implemented a prototype on Commercial Off The Shelf (COTS) hardware, replicating the Simple topology with load levels A, B, and C, which we describe next.

### 6.1. Implementation Setup

**Figure 11:**
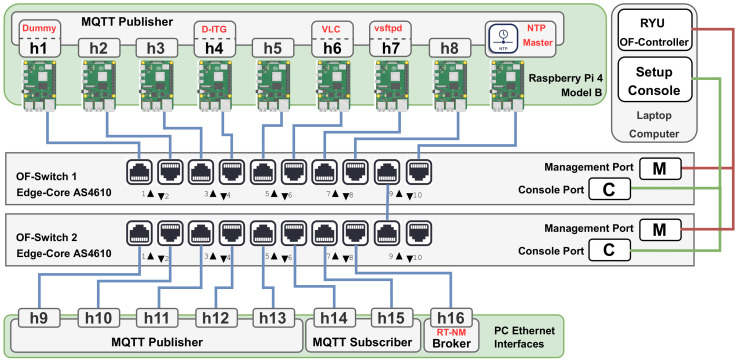
Experimental set-up architecture.

The hardware structure of the physical RT-MQTT set-up is shown in Figure 11. It comprises two Edge-Core AS4610-54P bare-metal switches that integrate Open vSwitch with OpenFlow support and one laptop computer equipped with a 4.9 GHz Intel Core(TM) I7 processor and 16 GB of RAM to run the SDN controller and a management console. Moreover, the MQTT Broker and eight MQTT clients are executed on one tower computer equipped with a 3.40 GHz Intel(R) Core(TM) i7-4770 processor, 8 GB of RAM and 10 individual Ethernet interfaces, 2 of which in the motherboard (Intel I210 Gigabit Network Connection) and 8 available via expansion cards (Intel Ethernet Server Adapter I350-T4). Finally, 8 Raspberry Pi 4 Model B are used to run additional MQTT clients. The software infrastructure is equivalent to the one used in emulation, i.e., the SDN controller is the RYU OF-Controller, Eclipse Mosquitto is used as the MQTT broker, and MQTT clients (Publishers and Subscribers) are based on the Eclipse Paho MQTT library.

One difference in the hardware implementation with respect to the emulation experiments is the absence of a global clock to carry out the latency measurements. To keep nodes synchronized, it was used the Network Time Protocol (NTP) [38]. A Raspberry Pi node, running the NTP Master exclusively and plugged into a dedicated port in the same switch as all other Raspberry Pi nodes, was added to the set-up. The synchronization period is set to a relatively short value (30 s) to minimize the impact of clock drift. Network delay jitter is minimized by assigning the highest priority to NTP transactions. Under these conditions, NTP is capable of sub-millisecond precision, which assures the significance of our measurements, particularly the maximum values observed, which are all in the range of 15 ms to 39 ms.

**Figure 12:**
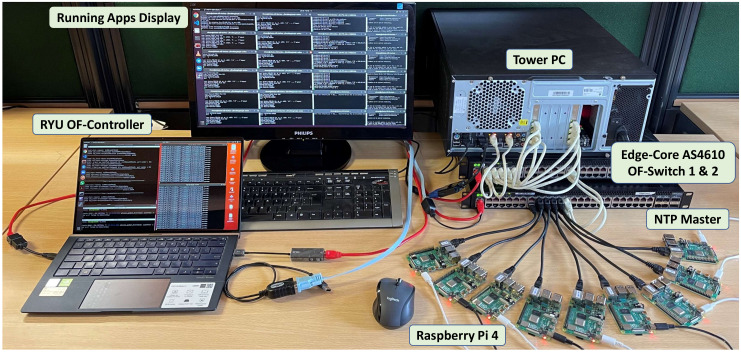
View of the complete experimental setup.

To follow closely the emulation scenario described in Section 5 the nodes were placed in equivalent positions and programmed to generate similar load patterns. For each load level, the experiments were also executed 200 times, with random placement of the publishers. Figure 12 shows the complete experimental set-up.

### 6.2. Experimental Results

**Figure 13:**
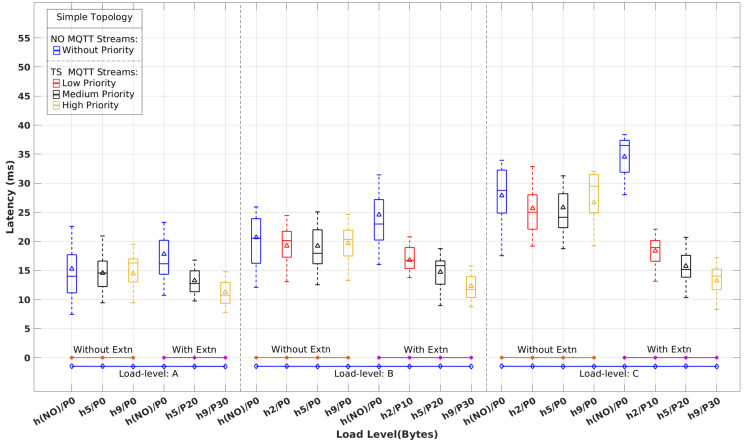
Latency of time-sensitive vs. normal MQTT flows with and without real-time extensions for the Simple topology physical implementation with load levels A, B, and C.

Figure 13 shows the latency distribution obtained for NO and TS flows, with and without the real-time extensions, considering just the Simple topology, as referred before, for the three load-levels {A, B, C}. The similarity to the corresponding emulation results shown in Figure 8 is striking. Apart from a slight increase in the observed absolute latency values for the TS flows in the physical experiment, all relative observations referred to before apply equally in this case.

The referred slight increase in latency is clearly visible by inspecting Table 5 and comparing it with the corresponding scenario in Table 3. This difference is essentially caused by the forwarding latency through the real switches, which is 3 ms to 4 ms higher than in Mininet. Moreover, the physical setup uses several publishers sharing the same computer, potentially causing mutual interference and consequent extra delay.

The results achieved with the physical setup establish the practicality of RT-MQTT using COTS equipment but also verify the emulation results, increasing their significance and showing the effectiveness and scalability of the proposed real-time MQTT extensions.

Table 6 shows the CI bounds for a 99% confidence level regarding the experiments with the physical implementation. The obtained CI bounds match the expected behavior growing with the load levels and decreasing with the priority. Compared with the CI bounds obtained in the emulation experiments (Table 4), the bounds obtained now with the physical implementation are higher. This is also expected, emerging directly from the higher latencies observed in the physical setup due to higher switch forwarding latency and computational resource sharing effects.

## 7. Conclusions and Future Work

Despite its increasing popularity in (I)IoT applications, MQTT doesn’t provide real-time services, impairing its use in scenarios that have timeliness requirements, such as those found in industrial environments. The literature presents several contributions that address this important limitation, but they are essentially focused on the broker’s performance. This work followed a more comprehensive approach, proposing a set of extensions to the MQTT protocol that allow specifying real-time requirements for time-sensitive flows while preserving full compatibility with the standard. Such specifications are then used by the network management, implemented in SDN/Openflow, to create real-time channels with suitable attributes. The extensions were implemented and subjected to an extensive performance assessment, both in emulation and physical setups. The results show the effectiveness of the time-sensitive traffic segregation and prioritization mechanisms, with significant latency reduction, particularly for higher network loads and complex topologies.

Future work includes developing analytic tools to estimate upper bounds for the time-sensitive traffic latency and its incorporation in the framework within an Admission Control service. Furthermore, complementary performance evaluation campaigns will be carried out, using concrete use cases and considering additional metrics, such as reliability, resource utilization, and scalability. In addition, the integration of security features will also be analyzed, considering both eventual conflicts, limitations, and impact on resource utilization and real-time performance. Finally, we also expect to implement a physical setup replacing SDN/Openflow with IEEE 802.1 TSN.

## Figures and Tables

**Table 1:** State-of-the-art characteristics summary.

Bib.	Prioritized	Network-level	Admission	Broker SW	Integ. management of TS
Reference	Traffic	Reservations	Control	Prioritization	Traffic (MQTT and Network)
[13]	Y	N	Y	E	Y
[14,15]	Y	N	Y	Y	Y
[16,17,18,19]	N	N	N	E	N
[20,21,22,23,24,25,26,29]	Y	Y	Y	NA	N
[27]	Y	Y	Y	NA	N
[28]	N	Y	N	E	Y
RT-MQTT	Y	Y	Y(FW)	E	Y

Y: Yes; N: No; E: Can be extended to allow; NA: Not Applicable; FW: Future Work.

**Table 2:** Emulation set-up parameters.

Parameters	Value
QoS level	1
Keep alive period	60 s
Traffic monitoring frequency	0.2 Hz
Publishing frequency	50 Hz
MQTT payload size	20 to 2000 Bytes
Time-sensitive publishing period	20±[0,0.4] ms
Maximum number of publications per publisher per test	1000

**Table 3:** Average/Maximum latency of TS and NO MQTT flows with real-time extensions.

		Latency (ms)					
		per Load-Level (A, B, C)					
		Avg			Max		
Topology	Publisher	A	B	C	A	B	C
Simple	h(NO)/P0	16	24	31	23	30	38
	h2/P10	×	13	15	×	20	21
	h5/P20	10	11	12	13	15	16
	h9/P30	8	10	11	11	12	13
Medium	h(NO)/P0	20	25	35	24	32	42
	h2/P10	×	15	16	×	20	21
	h5/P20	11	12	13	16	18	19
	h9/P30	10	11	12	15	16	18
Hard	h(NO)/P0	24	36	45	31	45	54
	h2/P10	×	18	20	×	22	24
	h5/P20	15	16	17	19	21	22
	h9/P30	13	14	16	19	20	21

**Table 4:** Confidence Intervals (99% confidence) for TS and NO MQTT flows: emulation.

		Latency Confidence Interval					
		Bounds (ms) per Load-Level (A, B, C)					
		A		B		C	
Topology	Publisher	LB	UB	LB	UB	LB	UB
Simple	h(NO)/P0	15.2	18.3	22.3	25.1	30.5	32.7
	h2/P10	×	×	12.5	14.0	14.3	16.0
	h5/P20	9.5	11.0	10.0	11.5	11.7	13.0
	h9/P30	7.5	8.5	9.5	10.8	10.7	11.3
Medium	h(NO)/P0	17.4	21.0	23.0	25.7	33.4	36.2
	h2/P10	×	×	14.0	15.6	15.0	16.7
	h5/P20	10.7	11.4	11.6	12.3	12.5	13.2
	h9/P30	9.1	10.4	10.2	11.1	11.0	13.0
Hard	h(NO)/P0	23.0	25.3	34.7	37.8	44.2	46.3
	h2/P10	×	×	17.6	18.4	19.7	20.3
	h5/P20	14.1	15.6	15.4	16.3	15.4	17.6
	h9/P30	12.0	13.5	13.6	14.4	15.8	17.1

**Table 5:** Average/Maximum latency of TS and NO MQTT flows with real-time extensions in the physical setup.

		Latency(ms)					
		per Load-Level (A, B, C)					
		Avg			Max		
Topology	Publisher	A	B	C	A	B	C
Simple	h(NO)/P0	18	25	35	24	31	39
	h2/P10	×	17	18	×	21	22
	h5/P20	13	15	16	17	19	21
	h9/P30	11	12	13	15	16	17

**Table 6:** Confidence Intervals (99% confidence) for TS and NO MQTT flows: physical setup.

		Confidence Interval Range of					
		Latency (ms) per Load-Level (A, B, C)					
		A		B		C	
Topology	Publisher	LB	UB	LB	UB	LB	UB
Simple	h(NO)/P0	17.1	18.4	24.0	25.7	34.2	36.0
	h2/P10	×	×	16.6	18.4	17.7	18.3
	h5/P20	12.6	13.7	14.5	15.3	15.4	16.3
	h9/P30	10.6	11.7	11.6	12.5	12.6	13.3

## Data Availability

Not applicable.

## Abbreviations

The following abbreviations are used in this manuscript:

IoT	Internet-of-Things
IIoT	Industrial-Internet-of-Things
MQTT	Message Queuing Telemetry Transport
QoS	Quality-of-Service
RT	Real-Time
SDN	Software-Defined Networking
RT-MQTT	Real-Time MQTT
COST	Commercial Off The Shelf
OF	OpenFlow
ONF	Open Networking Foundation
IntServ	Integrated Services
NV	Network Virtualization
PAC	Priority-based Admission Control
TS	Time-Sensitive
NO	Normal
OF-Switches	OpenFlow Switches
OF-Controller	OpenFlow Controller
OF-DB	OF-DataBase
RT-NM	Real-Time Network Manager
OVSDB	Open vSwitch Databas
e OVSDB-MP	Open vSwitch Database Management
D-ITG	Distributed Internet Traffic Generator
FTP	File Transfer Protocol
NTP	Network Time Protocol
